# Validity of additional surgical resection by comparing the operative risk with the stratified lymph node metastatic risk in patients with early gastric cancer after endoscopic submucosal dissection

**DOI:** 10.1186/s12957-019-1679-4

**Published:** 2019-08-05

**Authors:** Hidenori Akaike, Yoshihiko Kawaguchi, Kensuke Shiraishi, Hiroki Shimizu, Shinji Furuya, Naohiro Hosomura, Hidetake Amemiya, Hiromichi Kawaida, Makoto Sudoh, Shingo Inoue, Hiroshi Kohno, Daisuke Ichikawa

**Affiliations:** First Department of Surgery, Faculty of Medicine, University of Yamanashi, 1110 Shimokato, Chuo, Yamanashi, 4093898 Japan

**Keywords:** Additional gastrectomy, Early gastric cancer, Lymph node metastasis, National Clinical Database, In-hospital mortality

## Abstract

**Background:**

Treatment guidelines for early gastric cancer (EGC) recommend additional gastrectomy for lesions which do not achieve curative resection after ESD, due to the potential risk of lymph node metastasis (LNM). However, many cases are found to have no LNMs, and additional gastrectomy itself can be a considerable risk especially in elderly patients.

**Methods:**

We retrospectively stratified the risk of LNM according to the total number of four LNM risk factors (RFs) that resulted in non-curative resection for ESD in 861 EGC patients who underwent gastrectomy. Next, we compared this stratification risk to the surgical risk based on the National Clinical Database (NCD) risk calculator in 58 patients who underwent additional gastrectomy.

**Results:**

As the total number of LNM RFs increased, the frequency of LNM also increased significantly (0/1RF 0.76%, 2RFs 15.08%, 3RFs 33.87%, 4RFs 50.00%; *p* < 0.01). The estimated frequency of LNM was found to be lower than the predicted value of in-hospital mortality rate based on the NCD risk calculator in 25.0% of 0/1RF patients.

**Conclusion:**

These findings indicate, at least, that we should discuss the indication of additional gastrectomy individually for each patient from both perspectives of LNM and surgical risks.

## Background

Recently, endoscopic treatment for early gastric cancer (EGC) has been widely performed due to the improvement of diagnostic ability and progress of surgical procedures and medical devices [1]. The advent of endoscopic submucosal dissection (ESD) not only allowed accurate histopathological diagnosis but also reduced the local recurrence rate for even lesions larger than 2 cm and/or with ulcer scar which were difficult with conventional endoscopic mucosal resection (EMR) [2–4].

According to the guidelines for the treatment of gastric cancer, an absolute indication of ESD against EGC is defined as being intra-mucosal carcinoma and differentiated type without ulcer formation regardless of tumor diameter and also 3 cm or less in intra-mucosal carcinoma and differentiated type with ulcer formation. More recently, the indication has been expanded to lesions 2 cm or less of intra-mucosal undifferentiated carcinoma without ulcer formation following the results of clinical trials [5–7]. On the other hand, lesions, which do not meet the criteria for curative resection in the histopathological examination after ESD, are deemed to have a potential risk of lymph node metastasis (LNM), and additional gastrectomy with lymph node dissection (LND) is principally recommended only in terms of metastatic risk [7]. In recent years, however, the number of elderly patients has increased, having severe comorbidities, and an additional gastrectomy itself can be a considerable risk in such patients. In fact, it must also be recognized that there are many cases without metastasis in the retrieved lymph nodes even when additional gastrectomy with LND is performed.

Shoda et al. previously reported the usefulness of stratified LNM risk factors (RFs) for patients with EGC who did not meet absolute endoscopic resection in a single institution [8]. In the first step of this study, we reanalyzed the stratified LNM RFs by adding our case series. In the second step, we calculated a 30-day surgical death and hospital death risks of cases in which additional gastrectomy with regional LND was actually performed after ESD and assessed the validity of additional gastrectomy by comparing the operative risks with the stratified LNM risk in each case.

## Materials and methods

### Patients

We retrospectively analyzed EGC patients without treatment history diagnosed by pathological examination after being diagnosed by R0 gastrectomy. All of these patients underwent LND according to the gastric cancer treatment guidelines of the Japanese Gastric Cancer Association [7]. We added 385 EGC patients who underwent gastrectomy from 2005 to 2017 at the University of Yamanashi Hospital, to the 780 EGC patients who underwent gastrectomy from 1997 to 2014 at the Kyoto Prefectural University of Medicine Hospital. In total, of these 1165 patients, 861 EGC patients (571 in Kyoto series and 290 in Yamanashi series) whose tumors did not meet the criteria for curative endoscopic resection in the gastric cancer treatment guidelines [7] were enrolled in this retrospective study. Of this study group, 743 patients underwent radical surgery without EMR/ESD and 118 patients underwent additional gastrectomy after EMR/ESD.

**Fig. 1:**
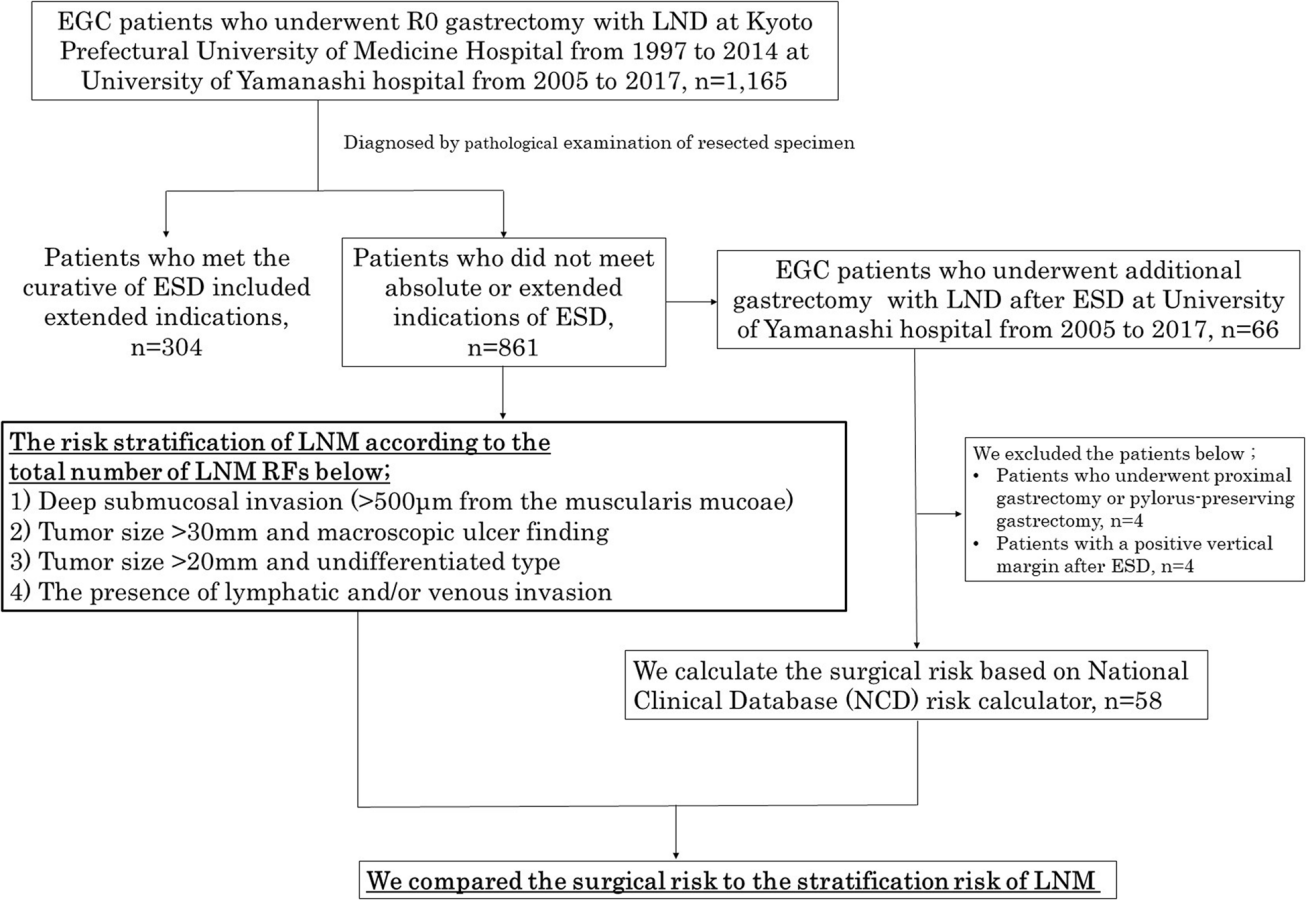
Flowchart of early gastric cancer patient enrollment in this study. EGC early gastric cancer, LND lymph node dissection, LNM lymph node metastasis, RF risk factor

Next, to assess the validity of additional gastrectomy after ESD, we enrolled 58 EGC patients who underwent ESD followed by additional gastrectomy with regional LND under the diagnosis of non-curative resection from 2005 to 2017 at the University of Yamanashi hospital. Patients who underwent limited partial resection of the stomach, such as partial local resection, proximal gastrectomy, and pylorus-preserving gastrectomy, and also patients with a pathological vertical positive margin after ESD were excluded (Fig. 1).

All procedures carried out in this study were in accordance with the ethical standards of the institutional and national responsible committee on human experimentation and the Helsinki Declaration of 1964 and its later amendments or equivalents. This study was approved by the University of Yamanashi Faculty of Medicine Ethics Committee. Informed consent was obtained from all individual patients included in the study.

### Lymph node metastasis risk factors (LNM RFs)

We defined the four items (see Fig. 1 for details) as LNM RFs for additional gastrectomy after EMR/ESD for EGC as reported by Shoda et al. [8] And we examined the LNM RFs and lymph node metastatic status in all of 861 patients and also examined whether the metastatic risk can be stratified by the total number of RFs as previously reported.

### National Clinical Database risk calculator

National Clinical Database (NCD) is a large-scale database project in Japan and established mainly by Japanese surgical academic societies. Japanese gastroenterological surgeons register all surgical patients and their detailed patients’ clinicopathological data on the website. The NCD was used to construct risk models for cancer-related surgeries, such as total and distal gastrectomy, etc., in Japanese patients, and the risk models have been already reported [9–11]. The risk models have been available as the “risk calculator” on the website, and the predicted 30-day mortality and in-hospital mortality can be calculated for each patient by entering clinical various data through the Internet. In this study, we used NCD risk calculator to evaluate surgical mortality, and compare the surgical risk with the risk of LNM in each patient with EGC.

### Statistical analysis

Statistical analysis was carried out using the statistical computing software R version 3.4.4 (R Foundation for Statistical Computing, Vienna, Austria).

Fisher’s exact test, chi-square test, Mann-Whitney *U* test, or Kruskal-Wallis test was used for statistical analyses. *p* values lower than 0.05 were considered statistically significant in all statistical tests.

## Results

### Stratification risk of LNM in EGC

The mean age of the patients was 65.77 years (± 11.06), and the gender ratio was 2.05:1 (male/female). The mean tumor size was 34.47 mm (± 20.54). The clinicopathological characteristics of patients by the total number of LNM RFs are shown in Table 1. The patients with histologically differentiated and smaller tumor size had fewer total numbers of LNM RFs. The patients with deeper tumors, more macroscopic ulcerations, or microscopically lymphatic and/or venous invasion were also fewer LNM RFs (Table 1).

**Table 1:** Comparison of clinicopathological characteristics according to the number of LNM risk factors in patients whose tumors did not meet the criteria for endoscopic resection

	*n*	Total number of items				*p* value
		0/1	2	3	4	
Total	861	396	305	124	36	
Age, mean ± SD (years)	65.77 (11.06)	64.69 (11.36)	66.14 (10.72)	68.01 (10.84)	66.72 (10.30)	0.024
Sex, *n* (%)						
Male	579 (67.2)	253 (63.9)	220 (72.1)	85 (68.5)	21 (58.3)	0.081
Female	282 (32.8)	143 (36.1)	85 (27.9)	39 (31.5)	15 (41.7)	
Tumor size, mean ± SD (mm)	34.47 (20.54)	28.71 (17.78)	35.14 (20.35)	45.47 (19.54)	54.31 (25.37)	< 0.001
Depth of tumor, *n* (%)						
M	317 (36.8)	237 (59.8)	78 (25.6)	2 (1.6)	0 (0)	< 0.001
SM1	144 (16.7)	118 (29.8)	17 (5.6)	9 (7.3)	0 (0)	
SM2	400 (46.5)	41 (10.4)	210 (68.9)	113 (91.1)	36 (100)	
Histological type, *n* (%)						
Differentiated	359 (41.7)	169 (42.7)	142 (46.6)	48 (38.7)	0 (0)	< 0.001
Undifferentiated	502 (58.3)	227 (57.3)	163 (53.4)	76 (61.3)	36 (100)	
Lymphatic invasion, *n* (%)						
Negative	605 (70.3)	365 (92.2)	196 (64.3)	41 (33.1)	3 (8.3)	< 0.001
Positive	256 (29.7)	31 (7.8)	109 (35.7)	83 (66.9)	33 (91.7)	
Venous invasion, *n* (%)						
Negative	706 (82.0)	385 (97.2)	227 (74.4)	71 (57.3)	23 (63.9)	< 0.001
Positive	155 (18.0)	11 (2.8)	78 (25.6)	53 (42.7)	13 (36.1)	
Ulceration (scar), *n* (%)						
Negative	196 (22.8)	104 (26.3)	72 (23.6)	20 (16.1)	0 (0)	0.001
Positive	665 (77.2)	292 (73.7)	233 (76.4)	104 (83.9)	36 (100)	
Preoperative ERM/ESD, *n* (%)						
Negative	743 (86.3)	337 (85.1)	256 (83.9)	114 (91.9)	36 (100)	0.012
Positive	118 (13.7)	59 (14.9)	49 (16.1)	10 (8.1)	0 (0)	

The LNM was present in 12.66% of all patients in this study (109/861, 95% confidence interval; 10.51%–15.07%). The frequencies of LNM stratified by the total number of four RFs are shown in Table 2. The frequency of LNM was significantly lower in patients with fewer LNM RFs. A significant correlation was found between the total number of LNM RFs and the frequency of LNM (*p* < 0.001). In particular, the frequency of LNM was far lower in patients with 0/1 LNM RFs, which was 0.76% (3/396, 95% confidence interval; 0.16–2.20), compared to the other groups (Table 2).

**Table 2:** Risk stratification of lymph node metastasis according to the total number of items that meet the indication criteria for radical gastrectomy

Total number of LNM risk factors	*n*	LNM positive cases, *n*	LNM rate, % (95% CI)	*p* value
Total	861	109	12.66 (10.51–15.07)	
Total number of items				
0/1	396	3	0.76 (0.16–2.20)	< 0.001
2	305	46	15.08 (11.26–19.60)	
3	124	42	33.87 (25.62–42.91)	
4	36	18	50.00 (32.92–67.08)	

### Comparison of the LNM risk and predicted surgical risk in cases which underwent additional gastrectomy for non-curative resection of ESD

The mean age of the patients was 67.4 years (± 9.9), and the gender ratio was 3.83:1 (male/female). There were 35 patients who received distal gastrectomy and 23 patients who received total gastrectomy. Thirteen patients had grade 2 or more postoperative complication of the Clavien-Dindo classification (22.4%). Table 3 shows the clinicopathological characteristics by the number of LNM RFs in these patient groups. No patients had four LNM RFs in this cohort. Patients with more LNM RFs had deeper tumors with more venous and/or lymphatic invasion.

**Table 3:** Comparison of clinicopathological characteristics according to the number of LNM risk factors

	*n*	Total number of items			*p* value
		0/1	2	3	
Total	58	40	14	4	
Age, mean ± SD (years)	67.4 (9.9)	66.3 (10.8)	68.4 (6.9)	74.8 (7.4)	0.38
Sex, *n* (%)					
Male	46 (79.3)	35 (87.5)	8 (57.1)	3 (75.0)	0.05
Female	12 (20.7)	5 (12.5)	6 (42.9)	1 (25.0)	
Tumor size, mean ± SD (mm)	27.5 (14.0)	26.5 (15.0)	27.8 (11.1)	36.8 (11.6)	0.38
Depth of tumor, *n* (%)					
M	8 (13.8)	8 (20.0)	0 (0)	2 (1.6)	< 0.01
SM1	22 (37.9)	19 (47.5)	2 (14.3)	1 (25.0)	
SM2	28 (48.3)	13 (32.5)	12 (85.7)	3 (75.0)	
Histological type, *n* (%)					
Differentiated	45 (77.6)	29 (72.5)	12 (85.7)	4 (100)	0.32
Undifferentiated	13 (22.4)	11 (27.5)	2 (14.3)	0 (0)	
Lymphatic invasion, *n* (%)					
Negative	36 (62.1)	31 (77.5)	4 (28.6)	1 (25.0)	< 0.01
Positive	22 (37.9)	9 (22.5)	10 (71.4)	3 (75.0)	
Venous invasion, *n* (%)					
Negative	44 (75.9)	34 (85.0)	9 (64.3)	1 (25.0)	0.05
Positive	14 (24.1)	6 (15.0)	5 (35.7)	3 (75.0)	
Ulceration (scar), *n* (%)					
Negative	35 (60.3)	27 (67.5)	6 (42.9)	2 (50.0)	0.24
Positive	23 (39.7)	13 (32.5)	8 (57.1)	2 (50.0)	
Type of gastrectomy, *n* (%)					
DG	35 (60.3)	24 (60.0)	9 (64.3)	2 (50.0)	0.87
TG	23 (39.7)	16 (40.0)	5 (35.7)	2 (50.0)	
Postoperative complication^a^, *n* (%)					
Negative	45 (77.6)	32 (80.0)	9 (64.3)	4 (100)	0.26
Positive	13 (22.4)	8 (20.0)	5 (35.7)	0 (0)	
30-day mortality, median range (%)	0.3 (0.1–6.3)	0.3 (0.1–6.3)	0.3 (0.1–0.4)	0.5 (0.2–0.6)	0.11
In-hospital mortality, median range (%)	0.5 (0.1–15.3)	0.5 (0.1–15.3)	0.5 (0.1–1.8)	0.7 (0.4–2.0)	0.59
Stratification risk of LNM, % (95% CI)	12.66 (10.51–15.07)	0.76 (0.16–2.20)	15.08 (11.26–19.60)	33.87 (25.62–42.91)	

^a^Clavien-Dindo classification grade 2 or more

Using the NCD risk calculator, the median in-hospital mortality was 0.5% (range; 0.1%–15.3%) in this study. There was no case in which the in-hospital mortality exceeded the stratified LNM frequency in the LNM RF 2 or 3 groups. However, the in-hospital mortality was higher than the stratified LNM frequency in some cases of the LNM RF 0/1 group (Table 3).

**Fig. 2:**
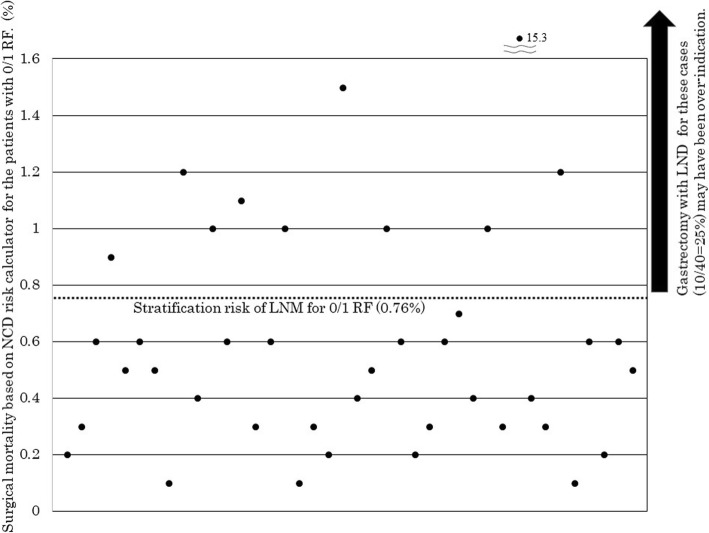
Surgical mortality based on NCD risk calculator for the patients with 0/1 risk factor. RF risk factor, LNM lymph node metastasis, LND lymph node dissection

As shown in Fig. 2, 10 cases showed higher in-hospital mortality than 0.76% which was a stratified LNM frequency in this group.

## Discussion

Previous reports demonstrated that the LNM frequency of gastrectomy with LND for cases diagnosed as non-curative resection by pathological examination after endoscopic resection was 3.8–8.2% [12–14]. Some endoscopists and surgeons examined whether the LNM frequency could be stratified according to several clinicopathological metastasis-related factors. In previous reports, Shoda et al. demonstrated that the LNM frequency well correlated with the number of RFs defined by them, and LNM frequency was extremely low as 0.58% in the 0/1 RF group [8]. We reanalyzed the LNM frequency with the criteria reported in a total of 861 patients treated at two different institutions and confirmed similar results, and the LNM frequency of the lowest risk group with 0/1 RF was found to be as low as 0.76%. Hatta et al. also reported in a multicenter collaborative study showing the risk-scoring system for LNM after ESD that does not meet the current curative criteria. In their study, they counted lymphatic invasion, which was reported to be a frequent LNM factor [15–17], as 3 points and counted each of other factors associated LNM (tumor size > 30 mm, positive vertical margin, venous invasion, and submucosal invasion ≥ 500 μm) as 1 point, stratified according to the total number, and the frequency of LNM was examined. The results of this study also showed that the LNM rate of the “low group” with the lowest risk of LNM was 2.5%, extremely low [18].

In Japan, the gastrectomy-related mortality has been considered to be very low, so additional gastrectomy with LND is recommended without much consideration for cases with potential risk after non-curative ESD. Recently, a so-called real-world Japanese data based on NCD clarified that in-hospital mortality by gastrectomy was not so low, 1.2% in distal gastrectomy [10] and 2.3% in total gastrectomy [11]. The mortality rate would not be negligible although the frequency might be relatively lower only in cases with EGC than reported. In recent years, elderly patients with EGC have been increasing due to recent aging society and popularization of medical examination in Japan. The physiological functions of various organs are generally deteriorated and comorbidities as potential surgical risk often present in elderly patients [19, 20]. These findings prompted us to examine whether the surgical risk of additional gastrectomy may be higher than the LNM frequency in some patients with EGC after ESD. In this study, we calculated the surgical risk of each cases using the NCD risk calculator and compared the predicted mortality risk with the LNM potential risk stratified according to the number of RFs. As a result, the predicted surgical mortality was higher than the potential frequency of LNM in 25% of the case in the 0/1 RF group, which indicates the additional gastrectomy might be an over-indication. All of our case series after ESD were also applied to the “eCura system” [18] to verify the categories of LNM. As a result, 4% of patients were classified as “low group,” and the LNM risk was lower than the predicted surgery-related mortality in 2.5% (one patient) in our series. The difference of LNM risks between our results and eCura system may be due to the facts, firstly, that the evaluated risk factors of the eCura system included vertical positive margin and, secondly, that it did not include histological undifferentiated adenocarcinoma. Further nation-wide large trials should enable more accurate LNM risk diagnosis after ESD.

In this study, patients who underwent additional gastrectomy after ESD and whose surgical risk was assessed using the NCD risk calculator had no in-hospital death. However, 36.2% of patients have postoperative complications. Postoperative complications are known as recurrence and poor prognosis factors after surgery for gastric cancer [21, 22].In addition, postoperative complications were noted in 5 of 10 cases, and their surgical risk was higher than the frequency of LNM. We think that if they had not received additional gastrectomy, their prognosis might have been better.

On the other hand, 35 patients with EGC were observed which were treated by ESD without surgery under the diagnosis of non-curative resection from 2005 to 2017 at the University of Yamanashi Hospital. Among them, only one case had a recurrence in regional lymph nodes (2.9%), and he was in the group with 2RFs. There was no recurrence in the 0/1RF group with low risk of LNM.

There are some limitations in our study. First is that our stratified LNM risk model was constructed based on clinicopathological data of EGC resected surgically. Second, the review of the comparison of LNM frequency and the surgical risk was a small-scale retrospective examination at a single facility. In the future, we might compare the prognosis of patients with higher surgical risk than LNM risk between follow-up without additional treatment group and additional gastrectomy group with regional lymph node dissection.

In conclusion, the present study clearly demonstrated that the LNM frequency could be stratified by the total number of the LNM RFs for the patients who do not meet the curative criteria after ESD. Furthermore, the risk comparison study suggested that there is a considerable number of cases in which their surgical risk was higher than the LNM risk among cases which additional gastrectomy was recommended for potential LNM risk. These findings indicated, at least, that we should discuss individually the indication of additional gastrectomy after ESD for each patient from both perspectives of LNM and surgical risks.

## Data Availability

Not applicable for this manuscript.

## Abbreviations

EGC: Early gastric cancer
EMR: Endoscopic mucosal resection
ESD: Endoscopic submucosal dissection
LND: Lymph node dissection
LNM: Lymph node metastasis
NCD: National Clinical Database
RF(s): Risk factor(s)
